# Immune Cell Landscape in the NSCLC Tumour Microenvironment: Molecular Dysregulation, Crosstalk and Insights into the CEMIP-STAT3-Mediated Immunosuppressive Axis

**DOI:** 10.3390/cancers18213451

**Published:** 2026-10-26

**Authors:** Lanqing Wang, Sisi Yan, Fajian He, Conghua Xie, Rui Bai

**Affiliations:** 1 Department of Radiation and Medical Oncology, Hubei Key Laboratory of Tumour Biological Behaviors, Hubei Cancer Clinical Study Center, Zhongnan Hospital of Wuhan University, Wuhan 430071, China; hustwlq1994@outlook.com (L.W.);; 2 Hubei Key Laboratory of Tumour Biological Behaviors, Zhongnan Hospital of Wuhan University, Wuhan 430071, China

**Keywords:** non-small cell lung cancer, tumour microenvironment, immune cell subsets, molecular mechanisms, signalling pathways, CEMIP, immunotherapy

## Abstract

Immunotherapy using immune checkpoint inhibitors has yielded meaningful clinical benefits for patients diagnosed with non-small cell lung cancer (NSCLC). Many patients still develop primary or acquired treatment resistance, a problem strongly tied to immunosuppressive features of the tumour microenvironment (TME). Multiple immune cell populations within the TME, including tumour-associated macrophages, CD8^+^ cytotoxic T cells, regulatory T cells, Natural killer (NK) cells and myeloid-derived suppressor cells, interact via soluble mediators and cell-surface molecules. Disordered crosstalk between these cell populations promotes tumour immune escape and limits immunotherapy effectiveness. In this review, we summarise major molecular mechanisms that control immune-cell behaviour in the NSCLC TME, placing special emphasis on the Cell migration inducing hyaluronidase 1 (CEMIP)-signal transducer and activator of transcription 3 (STAT3) axis described in pre-clinical work. Tumour-produced CEMIP is reported to promote macrophage polarisation toward an M2-like suppressive phenotype, putatively via an unidentified surface receptor and downstream STAT3 activation. This phenotypic shift in turn indirectly upregulates several inhibitory receptors on CD8^+^ T and Natural killer (NK) lymphocytes, offering a reasonable upstream mechanistic explanation for resistance to immune-checkpoint-blockade treatment. The supposed CEMIP receptor on monocytes has not yet been identified, and the possibility of indirect effects cannot be completely ruled out. For example, ECM remodeling or intermediary cellular signalling mediated by CEMIP is a possibility; for specific discussions. These unresolved mechanistic details, together with the lack of large-scale human NSCLC cohort validation, underscore that the CEMIP-STAT3 axis remains a pre-clinical observation requiring substantial further investigation before clinical translation can be contemplated. We therefore position this review as a critical, evidence-based assessment of the CEMIP–STAT3 hypothesis, rather than as a claim that this axis is an established driver of NSCLC immunosuppression.

## Simple Summary

Immunotherapy benefits NSCLC patients, but the immunosuppressive tumour microenvironment drives frequent immunotherapy resistance. This review describes major immune-cell populations in the NSCLC tumour microenvironment and their crosstalk, focusing on the pre-clinical CEMIP-STAT3 immunosuppressive axis, which reshapes macrophages and indirectly induces inhibitory-receptor-driven lymphocyte dysfunction. We summarise unresolved controversies, translational bottlenecks and prospective therapeutic strategies. We emphasise that CEMIP evidence is limited to pre-clinical studies without large-scale human NSCLC cohort validation. The main contribution of this review is an evidence-based evaluation of the proposed CEMIP–STAT3 axis in the NSCLC immune microenvironment; the immune-cell landscape is summarised as the necessary background for this assessment.

## 1. Introduction

Globally, lung cancer ranks first among cancer-associated causes of death. Roughly 85% of lung-cancer cases fall under the NSCLC histological subtype, which accounts for the majority of patient mortality linked to this malignancy [1,2]. Conventional therapeutic options such as chemotherapy, radiation therapy and molecular-targeted drugs have advanced considerably in recent years [3]. Still, patients diagnosed at the advanced stage gain very limited survival benefit; fewer than 20% of these patients can reach the five-year survival endpoint [4]. Immunotherapy relying on immune checkpoint inhibitors (ICIs) has opened new therapeutic avenues and extended survival for a subset of advanced-stage NSCLC patients. Nevertheless, ICI monotherapy can produce long-lasting anti-tumour effects only within 30–50% of unselected patient cohorts [5]. Quite a few patients exhibit primary tumour resistance with no measurable lesion reduction, or experience disease recurrence following transient tumour control. These practical clinical limitations narrow down the real-world gains brought by immunotherapy and create substantial obstacles for routine NSCLC clinical care.

Many early descriptions framed the tumour microenvironment (TME) as passive stromal tissue whose sole function was to support tumour growth. This view is incomplete, because the TME operates as a dynamic interactive compartment where tumour cells engage in complex signalling exchanges with diverse resident cell populations. These components encompass infiltrating immune cells, cancer-associated fibroblasts (CAFs), vascular endothelial cells and, extracellular matrix (ECM) elements, plus abundant secreted signalling molecules and metabolic intermediates. Joint activity among these TME-resident components influences multiple stages of tumour progression, ranging from primary tumour establishment and local tissue invasion to distant metastasis, tumour immune escape, and therapeutic responses against different anticancer regiment [6].

Various immune cell subsets serve as key constituents within the NSCLC TME. The balance between local anti-tumour immunity and immunosuppressive status is heavily determined by immune-cell infiltration profiles, polarisation phenotypes, cellular functional competence and cross-talk between distinct immune populations [7]. Growing clinical and experimental evidence demonstrates that functional impairments occurring in Tumour-associated macrophage (TAMs), CD8^+^ cytotoxic T cells, NK cells, Regulatory T cell (Tregs) and Myeloid-derived suppressor cells (MDSCs) bear close associations with patient prognosis and ICI therapeutic performance [8].

Over the past ten years, accumulating research work has expanded our knowledge regarding molecular events governing immune-cell behaviour inside NSCLC lesions. Cytokines, chemokines and metabolic signals, together with core intracellular cascades including STAT3, Hypoxia-inducible factor 1α (HIF-1α) and Phosphatidylinositol 3-kinase-Protein Kinase B signalling pathway (PI3K/Akt), cooperatively govern immune-cell differentiation and effector functions [9] CEMIP shows high expression in NSCLC clinical samples [10], STAT3-related signalling pathway has been identified as a newly described modulator for TAM polarisation [11], although direct experimental evidence for this link in lung cancer is still lacking. These observations have made CEMIP a candidate target for strategies aiming to reverse local immunosuppression [12].

Nevertheless, numerous knowledge gaps and conflicting experimental findings still remain unresolved. The complex molecular networks mediating immune-cell cross-talk have not yet been fully deciphered. Converting mechanistic discoveries obtained from bench-side experiments into practical clinical treatment schemes also poses considerable challenges. The present review summarises the latest advances in the molecular regulation of key immune subsets in the tumour microenvironment of non-small cell lung cancer and draws upon earlier studies, which laid the mechanistic foundation for current theories.

Both well-characterised signalling cascades and recently discovered regulatory mediators are included in our discussion. We aim to assemble molecular interaction schematics for immune-cell communication and interpret how compromised immune function can support prognosis assessment and ICI response forecasting. Unresolved scientific questions, ongoing academic disputes and viable future research avenues are also summarised. Using CEMIP as one representative emerging regulatory molecule, we analyse its biological roles inside the NSCLC TME and evaluate its translational prospect as a therapeutic target [13]. Focusing on underlying molecular mechanisms, this paper links pre-clinical experimental outcomes to real-world clinical scenarios and intends to offer practical references for fundamental and clinical research on NSCLC immunotherapy. The preclinical literature offers many appealing immune-related targets for NSCLC, yet validation in actual patients is scarce, and moving these strategies into the clinic has proven difficult. Our review therefore tries to keep one foot in the mechanistic biology and the other in the practical challenges of bringing these approaches to patients.

Growing clinical evidence indicates that elevated expression of inhibitory receptors (Programmed cell death 1 (PD-1), T-cell immunoglobulin and mucin-domain containing protein 3 (TIM-3), Lymphocyte-activation gene 3 (LAG-3), T-cell immunoreceptor with Ig and ITIM domains (TIGIT)) on tumour-infiltrating CD8^+^ T and NK cells marks a key phenotype linked to immune escape and unsatisfactory immune-checkpoint-blockade outcomes in NSCLC. Receptor upregulation is not solely governed by intrinsic cellular programming. Complex cytokine inputs and upstream microenvironmental stimuli from the TME also partially shape this molecular profile. In this review, we explore how the tumour-originated CEMIP-STAT3 axis reshapes local immune-cell populations and indirectly fuels immune suppression driven by these inhibitory receptors. Considering the pressing clinical need for new approaches to treat immunotherapy-resistant disease, we further examine pharmacological prospects for targeting proposed TME signalling hubs exemplified by CEMIP-STAT3.

Search Strategy: We searched PubMed and Web of Science up to September 2026 using combinations of terms covering NSCLC, the tumour microenvironment, individual immune-cell populations (macrophages, T cells, NK cells, B cells, myeloid-derived suppressor cells) and the signalling pathways discussed here (STAT3, NF-κB, PI3K/AKT, Programmed cell death ligand 1(PD-L1)). A separate search covered CEMIP, KIAA1199 and HYBID (alternative names for CEMIP). We prioritised studies from the past five years, but kept older papers when they established core mechanisms that later work builds on. Because this is a narrative review, no formal systematic search protocol or meta-analysis was used.

## 2. The Immune Cell Landscape in the NSCLC Tumour Microenvironment: Molecular Mechanisms of Dysregulation and Crosstalk

Tumour-mediated immune suppression in NSCLC cannot be attributed to any single immune-cell subset. This suppressive tissue phenotype arises from dynamic bidirectional signalling crosstalk among different immune populations, which is modulated by a broad set of soluble mediators and intracellular signalling modules. Importantly, most of these cell-to-cell communications operate via mutual feedback loops instead of unidirectional signal transfer, which serves to lock in an immune environment favourable for tumour growth.

This section describes key immune populations detected in human NSCLC tumour specimens, including their phenotypic features, functional defects, and core molecular regulatory frameworks. Beyond the independent biological behaviour of each immune subset, we also analyse how different immune groups interact to build interconnected signalling networks. We discuss well-characterised signalling cascades (STAT3, HIF-1α, PI3K/Akt) in breast cancer (evidence from other tumours’ immune mechanisms), alongside newly-identified regulatory molecules such as CEMIP [14]. Throughoutthis section, we outline how these molecular factors together shape the widespread immunosuppressive landscape observed in the NSCLC tumour microenvironment.

### 2.1. Tumour-AssociatedMacrophages (TAMs): The Core Regulator of Immune Suppression in NSCLC TME

**Figure 1:**
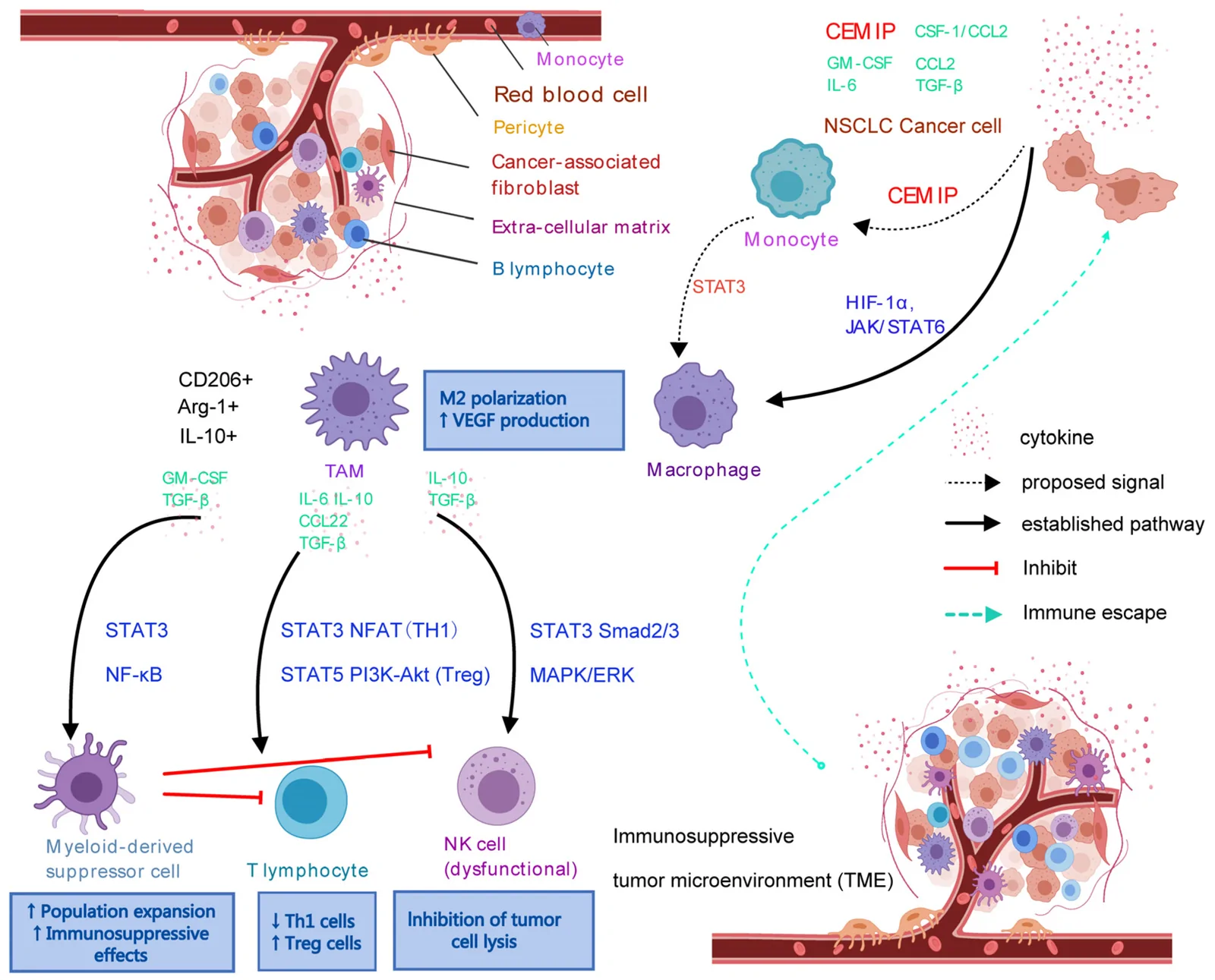
**The immunosuppressive tumour microenvironment of NSCLC.** NSCLC tumour cells secrete CEMIP, CSF-1/CCL2, GM-CSF, IL-6, CCL2 and TGF-β. These factors recruit monocytes into tumour tissue. Monocytes polarise into M2-like TAMs through STAT3, HIF-1α and JAK/STAT6 signalling. M2 TAMs express CD206, Arg-1 and IL-10 and secrete Vascular endothelial growth factor (VEGF). TAM-derived IL-10, TGF-β, IL-6 and CCL22 promote MDSC expansion, suppress Th1 cells, favour Treg accumulation and impair NK-cell function. The result is an immunosuppressive TME. Solid arrows denote established pathways. Dashed arrows denote proposed relationships. Red T-bars denote inhibition.

Within the NSCLC tumour microenvironment, tumour-associated macrophages (TAMs) represent the most plentiful immune-cell component, making up 30–50% of all immune cells present in tumour lesions in brain cancer [15], and TAMs are among the most abundant myeloid populations within the NSCLC tumour microenvironment [16]. They originate from circulating monocytes that are drawn into tumour sites through signalling molecules released by neoplastic and stromal cells alike. TAMs display considerable phenotypic and functional plasticity and can be divided into two major subgroups: anti-tumour M1-like macrophages and pro-tumour M2-like macrophages [17]. In patient-derived NSCLC tumour specimens, most TAMs shift toward an M2-skewed phenotype. Macrophages with this M2-biased profile drive tumour progression and metastatic spread and simultaneously weaken treatment responses to immunotherapy regimens [18]. Figure 1 illustrates the step-wise sequence starting from monocyte recruitment, proceeding to M2-type polarisation and culminating in local immunosuppressive outcomes.

#### 2.1.1. Phenotypic Characteristics of TAMs in NSCLC: M2 Polarisation Dominates the TME

Most studies of TAMs in NSCLC have relied on the M1/M2 dichotomy. This framework is useful but incomplete. Single-cell RNA sequencing has shown that macrophages in human lung tumours do not fall into two discrete states. Instead, they form a spectrum of transcriptional states, several of which coexist within the same tumour [19]. Some of these states carry distinct markers, such as Secreted phosphoprotein 1 (SPP1), Triggering receptor expressed on myeloid cells 2 (TREM2), Folate receptor 2 (FOLR2) or Macrophage receptor with collagenous structure (MARCO), and their abundance varies between patients [19,20]. In NSCLC, a MARCO-positive macrophage subset has been linked to immunosuppressive features and to co-expression of PD-L1 [20]. We therefore use the terms M1-like and M2-like throughout this review, while acknowledging that these labels simplify a more complex reality.

M1-polarised TAMs produce large panels of pro-inflammatory molecules, among which IFN-γ, Tumour necrosis factor α (TNF-α), IL-12, and chemokines C-X-C motif chemokine ligand (CXCL9) and C-X-C motif chemokine ligand 10 (CXCL10) stand out for their functional importance. Upon release, these molecules can activate CD8^+^ T and NK cell populations to mount potent anti-tumour immune reactions [21]. iNOS, CD86 and MHC-II are routinely used surface markers to define the M1 macrophage phenotype. By comparison, M2-biased TAMs release multiple immune-inhibitory molecules including IL-10 and TGF-β [22], as well aschemokines (C-C motif chemokine ligand 22 (CCL22), CCL17) and growth factors VEGF and Epidermal growth factor (EGF). Products secreted by M2-TAMs dampen the function of anti-tumour effector lymphocytes, support neoplastic cell proliferation, promote angiogenesis and help tumour cells escape immune surveillance. Researchers commonly apply CD206, Arg-1, IL-10 and CD163 to identify M2-like TAM subsets [23].

Retrospective patient-cohort studies have documented higher M2-TAM abundance within NSCLC tumour lesions relative to paired non-malignant peritumour tissues. Elevated CD206 expression, a standard marker for M2-type macrophages, correlates with poor tumour differentiation, lymph-node metastasis and reduced overall patient survival. Importantly, these statistical correlations do not equate to proven causal links. Tumours rich in iNOS-positive M1 macrophages, by contrast, are generally associated with better clinical prognoses [24]. Collectively, these clinical findings indicate that TAM polarisation profiles are key determinants shaping local immune balance inside NSCLC lesions.

#### 2.1.2. Molecular Mechanisms Driving TAM Polarisation in NSCLC

TAM phenotypic shifts inside the NSCLC tumour microenvironment arise from the intricate crosstalk of multiple molecular effectors and signal-transduction cascades. Multiple classes of regulators participate in this biological process, including cytokine-chemokine signalling axes, hypoxia-activated signalling events, and cellular metabolic reprogramming, together with newly-identified tumour-derived regulatory factors [25,26]. We summarise the core molecular mechanisms in the following sections:

(1) Cytokine/chemokine signalling pathways: Tumour and stromal compartments inside NSCLC lesions secrete a variety of cytokines and chemokines. These soluble factors recruit circulating peripheral monocytes into tumour sites and prime these precursor cells for M2-type differentiation [27]. Monocyte recruitment and priming are not driven by any single stimulus. Instead, combined signals derived from multiple different cell types regulate this biological process. Tumour-derived Colony-stimulating factor 1 (CSF-1) serves as a key mediator here: it binds to CSF1R expressed on monocyte surfaces and activates downstream PI3K/Akt signalling [28]. This signalling cascade increases transcript levels of M2-associated markers and blocks phenotypic reprogramming toward the M1-like macrophage phenotype [29]. In addition to signals released by cancer cells, intratumoural eosinophils and mast cells are capable of secreting IL-4 and IL-13. Once monocytes sense these cytokines, the JAK-STAT6 signalling cascade gets activated, pushing precursor cells further towards an M2-TAM-like phenotype. CCL2 secreted by NSCLC tumour cells additionally supports monocyte migration through the C-C motif chemokine ligand receptor 2 (CCR2)-Extracellular signal-regulated kinase (ERK) signalling cascade [30]. Chemotactic recruitment by itself is insufficient to complete full macrophage polarisation. It only builds a local microenvironment permissive for later M2-type phenotypic transformation.

(2) Hypoxia/HIF-1α signalling pathway: Among these markers, Arginase 1 (Arg-1) and iNOS are primarily established in mouse experimental systems; in human tissues, CD68, CD163 and CD206 are more commonly used to identify TAMs. Hypoxia constitutes one prominent hallmark within the NSCLC tumour microenvironment. When oxygen levels drop within tumour tissue, hypoxia-inducible factor-1α (HIF-1α) functions as the major molecular regulator driving TAM phenotypic reprogramming [31]. Hypoxia stabilizes intracellular HIF-1α protein in TAMs. This transcription factor directly binds the promoter regions of M2 marker genes (CD206 and Arg-1) and upregulates their transcription [32]. Meanwhile, HIF-1α inhibits the NF-κB signalling pathway and reduces the transcription of M1-related iNOS and TNF-α genes. Together, these transcriptional changes drive TAMs to adopt an M2-polarized phenotype [33].

(3) Metabolic reprogramming: Metabolic preferences in TAMs are more flexible in vivo than the simple M1-glycolysis/M2-FAO equation implies; the balance is shaped by the local environment rather than fixed by polarity. Metabolic reprogramming is a core molecular feature tightly linked to TAM phenotypic transformation. M2-polarized macrophages mainly obtain energy through fatty acid oxidation (FAO) and oxidative phosphorylation (OXPHOS). The upregulation of FAO-related enzymes, including Carnitine palmitoyltransferase 1A (CPT1A) and Acetyl-CoA carboxylase 1 (ACC1), is essential for monocyte transition toward the M2 phenotype [34]. Lactate released by NSCLC tumour cells suppresses monocyte glycolysis and shifts these immune cells toward FAO-dominated metabolic profiles, thereby promoting M2-type differentiation. Moreover, TME-derived Indoleamine 2,3-dioxygenase (IDO) triggers the kynurenine signalling cascade. This metabolic remodelling further reshapes macrophage metabolism and strengthens their immunosuppressive functions.

(4) The CEMIP-STAT3 axis: a proposed regulator of TAM polarisation

CEMIP is also known as KIAA1199 or HYBID. The protein is one of the few secreted factors with intrinsic hyaluronan-degrading activity. Overexpression has been reported across many epithelial tumour types [13,35].

The clinical evidence in NSCLC is consistent across several independent cohorts. CEMIP levels are higher in tumour tissue than in adjacent normal lung. High expression is associated with poor differentiation, lymph-node metastasis, advanced stage and shorter overall survival. One series of 153 NSCLC cases found strong CEMIP staining in 49.67% of tumours. Cox analysis identified CEMIP as an independent prognostic factor (*p* = 0.040) [36]. A second cohort confirmed the upregulation at both mRNA and protein levels, with an independent hazard ratio of 1.833 [10]. Database-based and tissue-based analyses in Lung adenocarcinoma (LUAD) and Lung squamous cell carcinoma (LUSC) reached the same conclusion [12,37].

These findings come from human tissue. Functional evidence points in the same direction. CEMIP binds Epidermal growth factor receptor (EGFR) and maintains EGFR-dependent phosphorylation of Src, Erk and Akt. It drives Epithelial-to-mesenchymal transition (EMT) in NSCLC cells, partly through the PI3K-Akt pathway [10,37]. Knockdown of CEMIP reduces xenograft growth in mice and improves the response to chemotherapy [10,37]. CEMIP also contributes to acquired drug resistance. ALKBH5-mediated demethylation upregulates CEMIP in paclitaxel-resistant cells [38]. CEMIP is also part of the non-canonical core transcriptional circuit that drives osimertinib resistance [39]. NF-κB induces CEMIP in non-lung systems. The protein then protects EGFR from degradation [40]. This point matters for NSCLC because NF-κB and EGFR are both constitutively active in a large proportion of tumours.

The link between CEMIP and STAT3 in NSCLC is much less secure. Not enough lung cancer study has so far shown that CEMIP activates STAT3. The closest evidence comes from breast cancer, where CEMIP promotes proliferation and migration through the Glucose-regulated protein 78 (GRP78)–STAT3 axis [14]. STAT3 itself is a well-established driver of M2 polarisation in macrophages [11].

CEMIP expression in human LUAD correlates with markers of TAMs (CCL2, CD68, IL-10), M2-like macrophages (CD163, V-set and immunoglobulin domain-containing protein 4 (VSIG4), Membrane-spanning 4-domains subfamily A member 4A (MS4A4A)), Tregs (Forkhead box P3 (FOXP3), C-C motif chemokine receptor 8 (CCR8), STAT5B, TGF-β) and exhausted T cells (CTLA-4, TIM-3) [12]. We do not interpret this correlation as evidence of causation.

**Figure 2:**
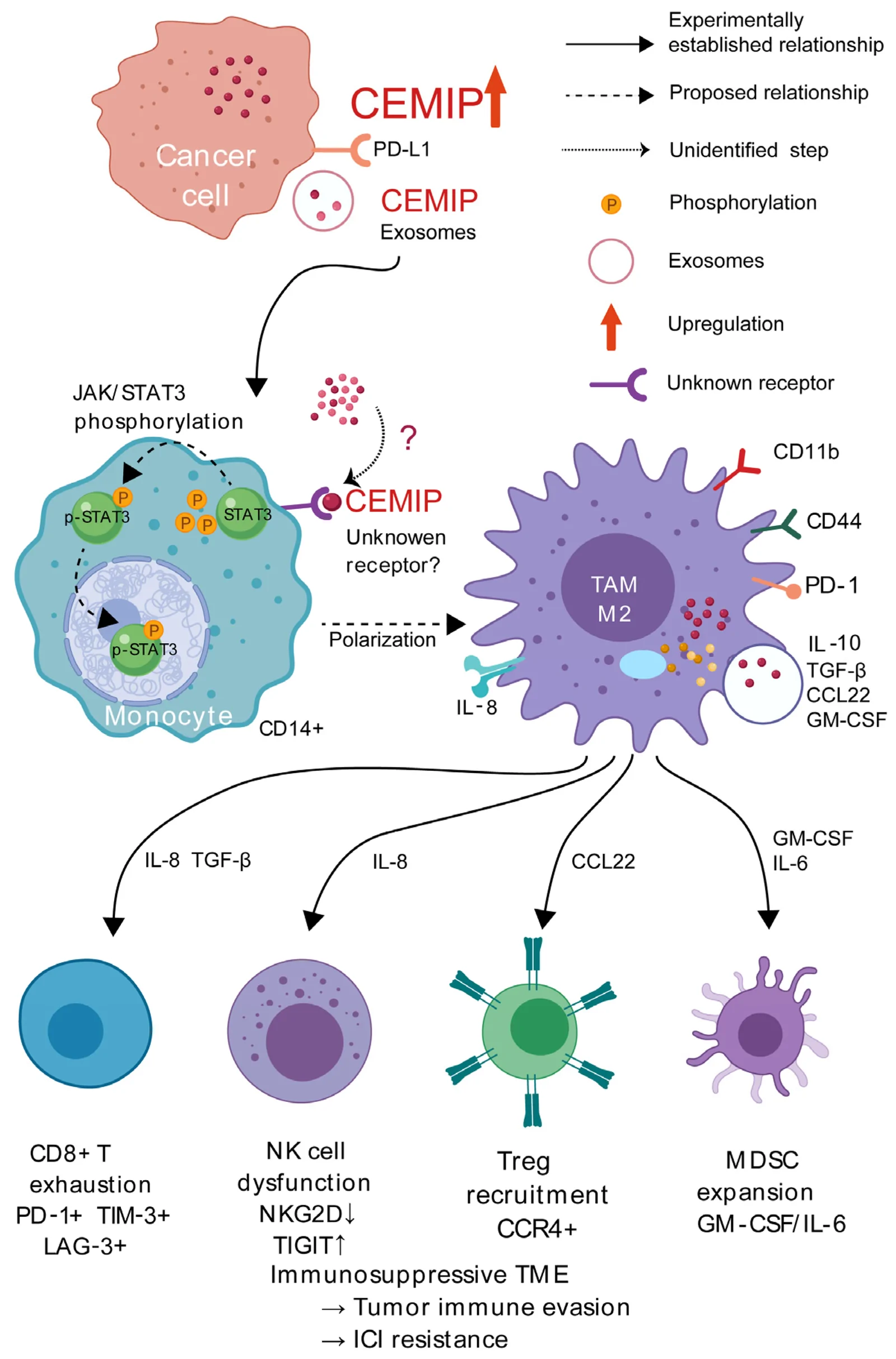
**Proposed CEMIP–STAT3 signalling in the NSCLC TME.** Cancer cells secrete CEMIP, which can also be carried by exosomes. CEMIP is presumed to act on an unidentified monocyte receptor (dotted line, question mark). Downstream JAK/STAT3 phosphorylation, p-STAT3 nuclear translocation and transcription of M2 genes (Arg-1, CD206) are shown as proposed steps. M2 TAMs express CD11b, CD44 and PD-1 and release IL-10, TGF-β, CCL22, GM-CSF and IL-6. These mediators drive CD8^+^ T-cell exhaustion, NK-cell dysfunction, Treg recruitment and MDSC expansion, culminating in immune evasion and ICI resistance. Solid, dashed and dotted arrows denote established, proposed and unidentified steps, respectively. P: phosphorylation. Upward arrow: upregulation. Dashed circle: unknown receptor.

The CEMIP–STAT3–TAM axis described below should therefore be read as a working hypothesis. Each step of the cascade rests on a different type of evidence. CEMIP is certainly secreted and remodels hyaluronan and the ECM. Its coupling to STAT3 has only been demonstrated outside the lung. STAT3-dependent M2 polarisation is well established in macrophages. The final step, CEMIP-dependent dysfunction of CD8^+^ T and NK cells, has not been tested. The monocyte/macrophage receptor for secreted CEMIP remains unidentified. Section 3.3 discusses alternative mechanisms that do not require such a receptor. The mechanistic framework underlying these opportunities is summarised in Figure 2.

#### 2.1.3. Clinical Significance of TAMs in NSCLC

TAM polarisation patterns and infiltration levels within the NSCLC tumour microenvironment correlate with patient clinical outcomes as well as ICI therapeutic performance. A meta-analysis [41] covering 20 studies including a total of 2,572 NSCLC patients reported that intratumoural M1 TAMs (CD68+Human leukocyte antigen (HLA-DR)+ or CD68+iNOS+) were strongly protective (Hazard ratio (HR) = 0.23, 95% Confidence interval (CI) 0.18–0.29, *p* < 0.00001), while stromal M2 TAMs (CD163+ or CD206+) conferred poorer survival (HR = 1.61, 95% CI 1.06–2.43, *p* = 0.02; for CD204+ M2 specifically HR = 2.01, 95% CI 1.43–2.82, *p* < 0.0001). These data support M2-TAM load as an independent adverse prognostic indicator for NSCLC. Within immunotherapy contexts, M2-type TAMs act as major drivers that give rise to ICI resistance. M2 TAMs can impair CD8^+^ T-cell anti-tumour competence through several distinct routes: releasing immunosuppressive cytokines IL-10 and TGF-β, expressing PD-L1 checkpoint protein, and triggering CD8^+^ T-cell exhaustion. This observation implies that M2-TAM infiltration magnitude may inform prognosis and warrant prospective validation as a predictive marker.

The impact of M2-like TAMs on checkpoint blockade extends beyond T cell suppression. In the lymphoma setting, PD-L1^+^CD163^+^ macrophages were shown to suppress PD-1^hi^ NK cells through the PD-1/PD-L1 axis, and PD-1 blockade restored NK activation in vitro. Given that M2-polarized TAMs in NSCLC frequently co-express PD-L1 and PD-L2, this mechanism provides a rationale for combining TAM-targeted strategies with PD-1/PD-L1 inhibitors in M2-enriched tumours [42]. Therapeutic approaches targeting TAM biology represent an appealing path to counteract TME-local immunosuppression and improve ICI effectiveness. Therapies targeting CSF-1R, including pexidartinib and cabiralizumab, can block monocyte recruitment and subsequent M2-TAM polarisation. These agents have yielded promising early-phase trial outcomes when combined with PD-1/PD-L1 immunotherapy [43]. Besides the inhibitors mentioned above, methods that promote the restoration of M2-like TAMs to an M1 phenotype include TLR agonists and Interferon γ (IFN-γ), and these clinical trials have already been conducted and summarised. Research shows that investigations targeting macrophages in cancer therapy have reviewed 606 clinical trials and 143 different tumour types, with TAM reprogramming being one of the main methods under evaluation [44]. However, for NSCLC, most of these works are still in preclinical or early clinical stages [44]. Figure 1 visually summarises the pro-tumour functions of M2-polarised TAMs in this context.

### 2.2. T Cell Subsets: The Core Force of Anti-Tumour Immunity and Its Molecular Dysregulation

T cell subsets are the core effector cells of anti-tumour immunity in the NSCLC TME, mainly including CD8^+^ cytotoxic T cells (CTLs), Tregs, and unconventional T cells (γδ T cells, Mucosal-associated invariant T (MAIT) cells) [45]. Their functional status is directly determined by a variety of molecular signals and signalling pathways in the TME, and their dysregulation is an important molecular basis for tumour immune escape and ICI resistance [46].

#### 2.2.1. CD8^+^ Cytotoxic T Cells (CTLs): Molecular Mechanisms of Exhaustion in the NSCLC TME

CD8^+^ cytotoxic T-lymphocytes serve as key effector mediators during tumour-targeted adaptive immunity. These lymphocytes recognise tumour-origin peptide antigens displayed by major histocompatibility complex (MHC-I) complexes via their T-cell receptor (TCR). Upon successful TCR engagement, CTLs release cytotoxic cargo containing perforin, granzyme B alongside IFN-γ to eliminate malignant tumour cells. Clinical observations have repeatedly linked abundant, functionally competent intratumoural CD8^+^ T-cell infiltration to improved patient prognosis. Exhaustion is not a single end state. CD8^+^ T cells progress along a differentiation continuum, from T cell factor 1 (TCF1)^+^ progenitor-like cells that retain proliferative and recall potential to Thymocyte selection-associated high mobility group box (TOX)^+^ terminally exhausted cells that have lost effector function [47,48], although initially defined in mouse models. The two populations differ in their response to checkpoint blockade: progenitor-like cells expand after anti-PD-1 treatment, whereas terminally exhausted cells respond poorly [47,48]. In NSCLC, single-cell sequencing has identified similar exhaustion states among tumour-infiltrating T cells, and the balance between them varies between patients [49]. Surface expression of PD-1, TIM-3, LAG-3 or TIGIT alone therefore does not define a terminally exhausted cell; the functional and transcriptional context matters. In a cohort of 303 resected NSCLC patients, high intratumoural CD8^+^ cell density was an independent favourable prognostic factor (relative risk (RR) = 0.37; *p* = 0.016) [50]. In comparison, patients with sparse intratumoural CD8^+^ T cell infiltration showed a five-year survival rate of merely 11.8%. Notably, this favourable prognostic correlation depends on the intact functional activity of infiltrated T cells. In most patients with advanced NSCLC, tumour-infiltrating T cells gradually lose their antitumour capacity despite successful tumour trafficking. This functional decay is widely termed T-cell exhaustion, and it largely explains why anti-tumour immunity often fails and why many patients respond poorly to ICI regimens [51].

(1) Phenotypic characteristics of exhausted CD8^+^ T cells: Exhausted CD8^+^ T cells within tumour tissues commonly upregulate multiple inhibitory surface receptors, including PD-1, TIM-3, LAG-3 and TIGIT. This receptor remodelling process [52] gradually impairs the effector functions of these lymphocytes. Such T cells generate fewer antitumour mediators such as IFN-γ, granzyme B and perforin, with compromised proliferative potential [53] and long-term survival ability. Importantly, inhibitory receptor expression does not follow a simple on-off pattern. Instead, the overall expression levels of these molecules reflect the severity of CD8^+^ T cell exhaustion [54], andthis molecular pattern is closely associated with clinical responses to immune checkpoint inhibition.

(2) Molecular mechanisms driving CD8^+^ T cell exhaustion: T-cell exhaustion within NSCLC lesions arises from interplay among diverse molecular mediators and signalling cascades. Two major sets of triggers contribute to this phenotype: persistent antigenic stimulation originating from tumour cells, plus suppressive inputs coming from the local TME [55].

(3) Chronic antigen stimulation and intrinsic signalling dysregulation: Continuous stimulation by tumour antigens maintains persistent activation of the TCR/CD3 signalling axis in CD8^+^ T cells [56]. This chronic signalling induces the upregulation of core transcription factors, such as TOX, Nuclear receptor subfamily 4 group A member 1 (NR4A1) and Nuclear factor of activated T cells (NFAT). Among these regulators, TOX is a dominant driver of T cell exhaustion. It remodels the epigenetic landscape of mature CD8^+^ T cells, preserving open chromatin at inhibitory receptor gene loci and sustaining the exhausted transcriptional profile [57]. In contrast, NR4A1 acts as a negative regulator of TCR signalling. It suppresses downstream NF-κB and Mitogen-activated protein kinase (MAPK) pathway activation, thereby attenuating the effector functions of CD8^+^ T cells.

(4) Immunosuppressive cytokines and signalling pathways: The immunosuppressive cytokines secreted by M2 TAMs, Tregs, and MDSCs in the NSCLC TME (IL-10, Transforming growth factor β (TGF-β), IL-6) are key extrinsic factors inducing CD8^+^ T cell exhaustion. IL-6 secreted by M2 TAMs activates the STAT3 signalling pathway in CD8^+^ T cells, upregulating the expression of PD-1 and TIM3 and inhibiting the secretion of IFN-γ [58]. TGF-β activates the Smad3 signalling pathway, which represses the transcription of effector genes (IFN-γ, granzyme B) and promotes the expression of inhibitory receptors. IL-10 inhibits the JAK/STAT1 signalling pathway in CD8^+^ T cells, impairing their anti-tumour function [59].

(5) Indirect regulation of CD8^+^ T cell exhaustion by CEMIP: No direct effect of CEMIP on T cells or NK cells has been reported so far. The available evidence is indirect and comes from three directions.

The first piece of evidence is correlational and comes from human tissue. CEMIP expression in LUAD is positively correlated with markers of TAMs, M2-like macrophages, Tregs and exhausted T cells [12]. The direction of this relationship is unknown.

The second piece comes from other tumour types. Exosomes from brain-metastatic breast cancer cells are enriched in CEMIP. These CEMIP-positive exosomes are taken up by microglia (the tissue macrophages of the brain) and by brain endothelial cells. They induce a pro-inflammatory programme (Ptgs2, Tnf, Ccl and Cxcl family members) and contribute to the formation of a metastatic niche [60]. Glioblastoma cells and tumour-associated macrophages both express HYBID/CEMIP. Depletion of CEMIP reduces macrophage infiltration and the release of pro-tumour factors [61].

The third piece of evidence is mechanistic. CEMIP degrades hyaluronan and thereby generates low-molecular-weight hyaluronan fragments. These fragments are known to signal through TLR2/TLR4 and CD44 and to modulate macrophage activation. This route would allow CEMIP to influence TAMs without a dedicated receptor or direct cell-cell contact [13,35]. CEMIP is also detectable in the extracellular vesicle fraction of plasma from lung cancer patients. Its levels are higher in patients with brain metastases [62].

These observations together support the following view. The sequence “tumour-derived CEMIP → unidentified monocyte receptor → JAK/STAT3 → M2 polarisation → cytokine-mediated CD8^+^ T/NK dysfunction” is a hypothesis rather than an established pathway. No study has tested it end-to-end in NSCLC. Whether CEMIP acts through a receptor, hyaluronan fragments, ECM remodelling or intermediary cells remains open. Section 3.3 outlines experiments that could distinguish these possibilities.

As such, this CEMIP-initiated, TAM-dependent signalling cascade represents one plausible upstream route through which CD8^+^ T cells may acquire an inhibitory-receptor-rich exhausted state within NSCLC tumours.

#### 2.2.2. Tregs: Molecular Mechanisms of Infiltration and Immune Suppression

Regulatory T cells (Tregs) form a specialised sub-population originating from CD4^+^ T cells. Foxp3 remains the master transcription factor that defines this lineage, alongside well-documented surface markers CD25, the α-chain of the IL-2 receptor, and CTLA-4 [63]. Tregs play a considerable part in building local immune-suppressive milieus within NSCLC tumour tissue. It should be kept in mind that these cells are not universally harmful; their actual biological output can shift according to tumour-site context. Tissue from NSCLC lesions frequently harbours higher Treg numbers relative to paired non-tumour adjacent samples, and raised Treg levels correlate with poorer prognosis and sub-optimal ICI therapeutic outcomes. In a cohort of patients with stage I–III disease, the density of FOXP3-positive cells were significantly higher in stage II tumours [64]. Treg abundance also affects how patients respond to checkpoint blockade. Kagamu et al. treated patients with nivolumab and found that non-responders had a higher baseline proportion of CD25^+^FOXP3^+^ cells among CD4^+^ T cells in the blood (*p* = 0.034) [65]. Zuazo et al. reported a similar picture in 83 patients with advanced NSCLC who received PD-1/PD-L1 inhibitors: patients with a low baseline Treg fraction had higher response rates and longer progression-free and overall survival (sensitivity 70%, specificity 100%) [66]. Together, these observations suggest that a high Treg fraction, either in the tumour or in the blood, is associated with a poorer response to immune checkpoint inhibitors. Such clinical differences highlight the significance of Treg biology in NSCLC. Multiple signalling cascades control the accumulation and suppressive activation of Tregs in tumour tissues. This phenomenon is primarily driven by two mechanisms: chemokine-dependent Treg recruitment and cytokine-triggered functional maturation [67].

(1) Chemokine-driven recruitment: Tumour cells and M2-polarised TAMs produce large quantities of CCL22. This chemokine interacts with CCR4 expressed on Tregs and draws circulating peripheral Tregs into tumour tissue through CCR4-dependent PI3K/Akt signalling [68]. CCL17, which is secreted mainly by dendritic cells and tumour cells, attracts Tregs through the same receptor [69].

(2) Cytokine-driven activation and functional strengthening: TGF-β and IL-2 released by tumour cells, M2 TAMs and MDSCs represent central regulators shaping Treg behaviour. When naïve CD4^+^ T-cells receive TGF-β stimulation [70], Smad3 and STAT5 become activated simultaneously [71]. Combined signalling pushes these cells to differentiate into peripheral inducible Tregs (iTregs) and increases Foxp3 gene transcription. IL-2 interacts with the multi-subunit IL-2 receptor complex (CD25/CD122/CD132) on Tregs and activates the downstream JAK-STAT5 signalling pathway [67]. This signalling axis sustains Treg proliferation, enhances cell survival, and strengthens their immunosuppressive properties.

(3) Metabolic regulation: The metabolic characteristics of the NSCLC tumour microenvironment, including glucose scarcity, lactate accumulation and tryptophan depletion, can profoundly modulate Treg function [72]. Compared with effector T cells, Tregs exhibit superior metabolic adaptability to hypoxic and nutrient-deprived tumour conditions [73]. Specifically, tumour cell-derived lactate activates the GPR81/ERK signalling pathway in Tregs, which further potentiates their immunosuppressive activity.

(4) Controversies in Treg function: The functional role of Tregs within the NSCLC tumour microenvironment remains debatable. Several reports indicate that Tregs dampen antitumour immunity by restraining CD8^+^ T-cell and NK-cell activities [74]. On the other hand, limited evidence suggests that some Treg subsets may carry out antitumour functions by modulating other immune cells. They can restrain the expansion of pro-tumour Th17 cells, an effect reported mainly in colorectal cancer [75]. These conflicting observations may reflect Treg heterogeneity, such as the distinction between thymic-derived (tTreg), peripherally induced (pTreg) and in vitro-induced (iTreg) Tregs, as well as their changing functional states across tumour stages and local microenvironments [76]. Further single-cell-based investigations are therefore required to dissect the precise functions of distinct Treg populations in NSCLC.

#### 2.2.3. Unconventional T Cells: γδ T Cells and MAIT Cells

γδ T cells and MAIT cells are unconventional T cells. They play unique roles in the anti-tumour immune response of the NSCLC TME. Their molecular regulatory mechanisms are gradually being elucidated.

(1) γδ T cells: γδ T cells can recognize tumour-associated antigens (e.g., phosphoantigens, MHC class I polypeptide-related sequence A/B (MICA/B)) in a major histocompatibility complex (MHC)-independent manner [77], and exert anti-tumour effects via the secretion of pro-inflammatory cytokines (IFN-γ, TNF-α) and direct tumour cell killing. The infiltration of γδ T cells in the NSCLC TME is positively correlated with patient prognosis [78], and the activation of γδ T cells via phosphoantigens or TCR agonists can enhance the anti-tumour immune response. In the NSCLC TME, γδ T-cell effector functions are governed by signals from the TCR and innate receptors, because the γδTCR–ERK axis promotes proliferation and cytokine production and NKG2D–DNAX-activating protein of 10 kDa (DAP10) driving degranulation and cytotoxicity [79]. In addition, γδ T cells display both anti-tumour and pro-tumour roles (Antitumour: Secretes IFN-γ and TNF-α; kills tumour cells via Fas receptor/Fas ligand (Fas/FasL) and ADCC; Pro-tumour: tumour-infiltrating γδ T cells are one of the main sources of IL-17, which promotes angiogenesis) depending on subset and local signals, and this duality has been reviewed extensively [80].

(2) MAIT cells: MAIT cells are largely located in mucosal tissues, where they detect bacterial and viral antigens presented by MR1 molecules [81]. In the NSCLC tumour microenvironment, infiltrating MAIT cells secrete IFN-γ and TNF-α to carry out antitumour functions, and the level of MAIT-cell infiltration is linked to how patients respond to immune checkpoint inhibitors. MR1/TCR and JAK/STAT1 signalling control MAIT-cell activity in this setting [82]. However, immunosuppressive cytokines such as IL-10 and TGF-β within the TME can hold back MAIT-cell activation and effector responses. Overall, existing studies on γδ T cells and MAIT cells in NSCLC remain quite limited. We still need more work to clarify their detailed molecular mechanisms and clinical relevance.

### 2.3. Other Immune Cell Subsets in NSCLC TME: Molecular Mechanisms of Dysregulation

In addition to TAMs and T cell subsets, NK cells, MDSCs, and B cells are also important immune cell subsets in the NSCLC TME, and their functional dysregulation via specific molecular mechanisms further exacerbates the immunosuppressive state of the TME [83].

#### 2.3.1. NK Cells: Molecular Mechanisms of Dysfunction

Natural-killer (NK) cells constitute key innate immune sentinels that recognise and dispose of malignant cells. Unlike T-cell-driven anti-tumour responses, NK-mediated cytotoxicity does not require prior antigen sensitisation. These immune cells release granules loaded with perforin and granzyme B to exert killing effects against tumour cells [84]. Clinical cohorts have associated well-preserved NK-cell activity with more favourable outcomes in NSCLC [85]. Once inside the tumour, however, tumour-infiltrating NK cells often show severely impaired cytotoxic function. It is worth emphasising that this dysfunction is not a uniform, on-or-off state; receptor profiles can shift gradually as local TME signals accumulate. Part of the functional impairment of NK cells in NSCLC reflects changes in their surface receptors. Activating receptors, including NKG2D, Natural cytotoxicity triggering receptors (NKp30) and NKp46, are downregulated, while inhibitory receptors such as Killer-cell immunoglobulin-like receptor (KIR), TIGIT and PD-1 are upregulated. In human NSCLC tissue, tumour-infiltrating NK cells express substantially less NKG2D than NK cells from the peripheral blood of the same patients or from healthy donors, and a similar reduction is seen for other activating receptors [86]. Low NKG2D levels have also been linked to worse prognosis and a higher risk of metastasis in some studies, although these associations are correlative rather than causal.

Molecular mechanisms of NK cell dysfunction in the NSCLC TME: The dysfunction of NK cells is mainly induced by the immunosuppressive [87] TME via multiple molecular signalling pathways:

(1) Immunosuppressive cytokine-mediated inhibition: M2 TAMs and Tregs in the NSCLC TME secrete IL-10 and TGF-β, which are key factors inhibiting NK cell function [88]. IL-10 activates the STAT3 signalling pathway in NK cells, downregulating the expression of activating receptors (NKG2D, NKp30) and inhibiting the secretion of IFN-γ. TGF-β activates the Smad2/3 signalling pathway [89], which represses the transcription of effector genes (perforin, granzyme B) and upregulates the expression of inhibitory receptors (TIGIT).

(2) MDSC-mediated inhibition: MDSCs in the NSCLC TME inhibit NK cell function via the production of reactive oxygen species (ROS) and nitric oxide (NO), and the depletion of arginine [90]. ROS and NO damage the DNA and proteins of NK cells, inhibiting the activation of the MAPK/ERK signalling pathway and impairing their killing ability [91]. Arginine depletion by MDSCs downregulates the expression of CD3ζ on the surface of NK cells, further inhibiting their activation [92].

(3) tumour cell-derived ligand downregulation: tumour cells in the NSCLC TME downregulate the expression of NKG2D ligands (MICA/B, ULBP) via proteolytic cleavage and epigenetic modification, reducing the recognition and activation of NK cells [93], which is an important molecular mechanism of tumour immune escape from NK cell surveillance.

#### 2.3.2. Myeloid-Derived Suppressor Cells (MDSCs): Molecular Mechanisms of Accumulation and Immune Suppression

Myeloid-derived suppressor cells (MDSCs) are a heterogeneous group of immature myeloid cells with strong immunosuppressive activity [94]. In NSCLC, their expansion in blood and tumour tissue has been linked to advanced disease, metastasis, and a poorer response to immune checkpoint inhibitors [95]. Two main subsets are recognised in humans: granulocytic MDSCs (G-MDSCs; CD11b CD33^+^HLA-DR CD15^+^) and monocytic MDSCs (M-MDSCs; CD11b^+^CD33^+^HLA-DR CD14^+^) [96]. G-MDSCs usually predominate, accounting for more than 80% of circulating MDSCs in most cancer types, although the exact proportion varies between studies and tumour sites. In a meta-analysis of 722 NSCLC patients, high circulating MDSC levels were associated with shorter progression-free, recurrence-free, and overall survival [95]. These associations are not strong enough to treat MDSC measurements as a validated prognostic biomarker, but MDSC load is also best regarded as a candidate biomarker for clinical assessment in NSCLC.

(1) Molecularmechanisms of accumulation: Tumour cells and stromal cells in the NSCLC TME release several factors including Granulocyte-macrophage colony-stimulating factor (GM-CSF), Granulocyte colony-stimulating factor (G-CSF), IL-6, and Prostaglandin E2 (PGE2). These factors drive MDSC expansion in the bone marrow and their mobilisation into the circulation [94]. MDSC are attracted to tumour sites along chemokine gradients: monocytic MDSCs follow the CCL2/CCR2 axis, whereas granulocytic MDSCs respond mainly to CXCL8/CXCR2 signalling. At the molecular level, IL-6 and GM-CSF activate STAT3 in myeloid progenitors. This pathway keeps these cells from differentiating into mature dendritic cells or macrophages and instead favours their expansion into suppressive MDSCs [97].

(2) Molecular mechanisms of immune suppression: MDSCs exert immunosuppressive effects on the anti-tumour immune response via multiple molecular mechanisms [98], which are the core drivers of the immunosuppressive TME:

A. Amino acid depletion: MDSCs express high levels of Arg-1 and IDO [99], which deplete arginine and tryptophan in the TME, respectively, inhibiting the proliferation and function of CD8^+^ T cells and NK cells [100].

B. ROS and NO production: MDSCs produce large amounts of ROS and NO via the Nicotinamide adenine dinucleotide phosphate (NADPH) oxidase [101] and inducible nitric oxide synthase (iNOS) pathways, which damage the TCR complex of T cells and inhibit the activation of the MAPK signalling pathway [102] thereby impairing T cell function.

C. Cytokine secretion: MDSCs secrete IL-10 and TGF-β [103], which further reinforce the immunosuppressive TME and promote the polarisation of TAMs to the M2 phenotype and the proliferation of Tregs [104].

D. Immune checkpoint molecule expression: MDSCs express PD-L1 on their surface, which binds to PD-1 on CD8^+^ T cells and induces T cell exhaustion via the PD-1/PD-L1 signalling pathway [105].

#### 2.3.3. B Cells and Tertiary Lymphoid Structures (TLS): Molecular Mechanisms and Controversies

B lymphocytes belong to adaptive immune compartments. They produce antibody molecules and take part in anti-tumour defence through humoral immunity alongside antigen-presenting activity. Within NSCLC tumour sites, B cells can assemble TLS together with T cells [106], dendritic cells (DCs) and stromal elements. TLS represent ectopic lymphoid-like tissue assemblies capable of boosting anti-tumour immune activation. Detectable TLS within NSCLC specimens correlates with stronger CD8^+^ T-cell infiltration and improved ICI treatment responses, making TLS a promising positive-prognosis marker for NSCLC patients.

Signalling controlled by the C-X-C motif chemokine ligand 13 (CXCL13)-C-X-C motif chemokine receptor 5 (CXCR5) chemokine pair as well as lymphotoxin (LT) cascades govern TLS assembly inside tumour tissue [107]. Tumour-associated DC populations and stromal cells release CXCL13. This chemokine attracts CXCR5-expressing B and T lymphocytes to build early TLS precursor structures. The LTα1β2-Lymphotoxin β receptor (LTβR) signalling axis then drives TLS maturation and germinal-centre generation [108]. Mature TLS support B-cell and T-cell proliferation and differentiation, therefore mounting effective anti-tumour immune activity.

Debates persist regarding overall B-cell functions within the NSCLC TME. Apart from their tumour-protective roles mediated by TLS formation, certain B-cell subgroups such as regulatory B cells (Bregs) can produce immune-suppressive IL-10 and TGF-β. Through CD40-CD40L signalling interactions [109], Bregs can also encourage Treg expansion and dampen anti-tumour immune activity. This difference may partly reflect the distinction between overall B-cell infiltration and mature, well-organized TLS. A favorable prognosis is primarily associated with mature TLS containing germinal centres, whereas diffuse or immature lymphocyte aggregates do not confer this prognostic advantage. Such conflicting observations likely arise from B-cell-subset heterogeneity, different TLS developmental stages and variable local TME conditions. Additional experimental work remains necessary to clarify molecular circuits shaping distinct B-cell-subset behaviour in NSCLC lesions.

### 2.4. Molecular Network of Immune Cell Crosstalk in the NSCLC TME

#### 2.4.1. Exosomes as a Route of Intercellular Communication

Exosomes carry proteins, lipids, mRNAs and microRNAs between cells. Tumour-derived exosomes can suppress antitumour immunity. Melanoma exosomes carry surface PD-L1, which inhibits CD8^+^ T cells, and circulating exosomal PD-L1 levels track with the response to anti-PD-1 therapy [110]. Exosomes also matter for the CEMIP axis. CEMIP is enriched in exosomes from brain-metastatic breast cancer cells, and uptake of these exosomes by microglia and brain endothelial cells induces a pro-inflammatory programme [60]. CEMIP is also detectable in the extracellular vesicle fraction of lung cancer patient plasma, with higher levels in brain-metastatic patients [62]. Secreted CEMIP may therefore act not only as a soluble protein but also via exosomal transport, although this route has not been examined in NSCLC.

#### 2.4.2. Crosstalk Among AKT, NF-κB and STAT3 Signalling and PD-L1 Regulation

AKT, NF-κB and STAT3 are often presented as separate cascades, yet their activities converge inside the NSCLC TME. TNF-induced NF-κB activation requires the Akt serine-threonine kinase, placing AKT upstream of IKK in this pathway [111]. STAT3 and NF-κB are both constitutively active in a large proportion of NSCLC tumours, and both contribute to PD-L1 regulation. STAT3 binds the CD274 promoter and drives PD-L1 transcription, as first demonstrated in ALK-positive T-cell lymphoma. At the same time NPM/ALK activates STAT3 and induces PD-L1 expression [112]. In NSCLC, c-MET-dependent NF-κB activation has been shown to modulate PD-L1 and CCL22 expression and to promote immune evasion [113]. Loss of PTEN increases PD-L1 protein through PI3K/AKT activation; this effect operates at the post-transcriptional level in glioma [114]. These routes are not mutually exclusive. In tumours, EGFR, PI3K/AKT, NF-κB and STAT3 are co-activated, they probably act together to maintain high PD-L1 expression and to sustain M2-like TAM polarisation. This redundancy has a direct implication for the CEMIP–STAT3 hypothesis discussed below: any CEMIP-dependent contribution would have to operate on top of these established upstream signals. Loss-of-function experiments are therefore required to establish a non-redundant role for CEMIP, as outlined in Section 3.3.

Immune subsets residing in the NSCLC tumour microenvironment rarely function in isolation. Cytokines, chemokines and intracellular signalling pathways connect distinct immune cell types into a highly interconnected regulatory network. Combined crosstalk between these cell populations builds local immunosuppressive conditions, which in turn promotes tumour progression and fosters ICI therapeutic resistance. Major molecular interactions are outlined in the following text.

Immunosuppressive phenotypes seen in NSCLC develop through feed-forward signalling cascades triggered by malignant tumour-cell-derived signals [3]. NSCLC tumour cells constitutively release a panel of bioactive molecules: CEMIP, CCL2, CSF-1, GM-CSF, IL-6 and TGF-β, which together reshape the local immune milieu. For instance, tumour-derived CCL2 and CSF-1 engage CCR2 and CSF1R receptors expressed on circulating monocytes. This signalling recruits monocytes into tumour tissue and pushes these precursor cells toward M2-type macrophage differentiation [115]. CEMIP is proposed to engage a yet-unidentified receptor present on monocyte membranes. This putative ligand-receptor engagement is hypothesised to launche intracellular signalling, inducing JAK-STAT3 phosphorylation followed by STAT3 translocation into the nucleus. Within the nucleus, STAT3 increases transcriptional output of M2-associated marker genes including Arg-1 and CD206 [116].

**Figure 3:**
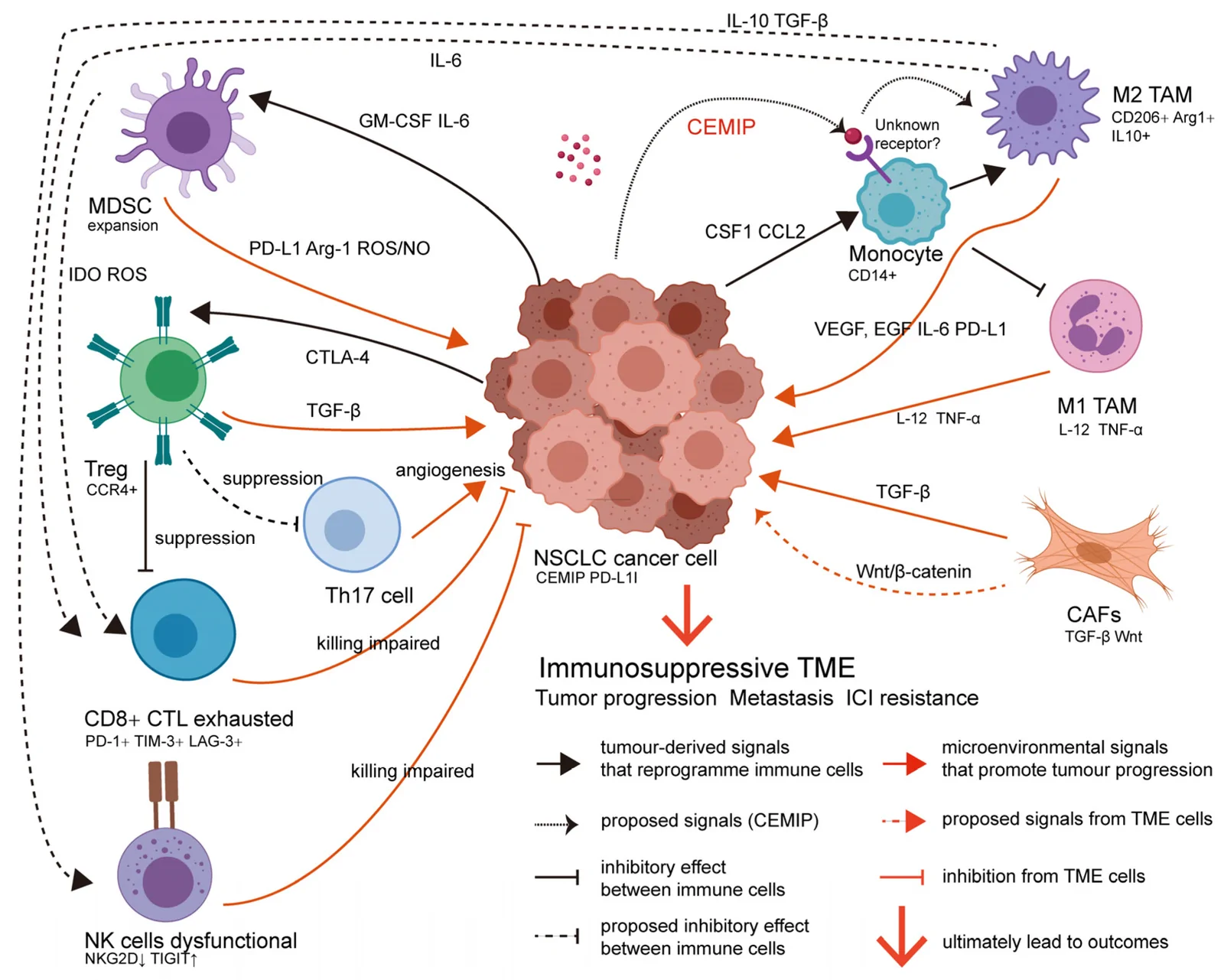
**Bidirectional crosstalk between NSCLC cells and immune cells in the TME.** Black arrows show tumour-derived signals that reprogramme immune cells. CSF1 and CCL2 recruit monocytes. CEMIP drives M2 polarisation (dashed line). GM-CSF and IL-6 expand MDSCs. TGF-β supports Treg accumulation. Red arrows show microenvironmental signals that feed back to tumour cells. TAMs release VEGF, EGF, IL-6 and PD-L1. MDSCs produce PD-L1, Arg-1 and ROS/NO. Tregs express CTLA-4. CAFs secrete TGF-β and Wnt/β-catenin. Th17-derived IL-17 promotes angiogenesis. Dysfunctional CD8^+^ T and NK cells fail to kill tumour cells (red T-bars). Together, these interactions create an immunosuppressive TME that drives tumour progression, metastasis and ICI resistance. Solid lines denote established relationships. Dashed lines denote proposed or extrapolated relationships.

Once monocytes differentiate into M2-TAMs, these macrophages become central signalling hubs that amplify local immune-inhibitory outputs. M2-TAMs secrete high levels of IL-6, IL-10, TGF-β and CCL22; each mediator executes discrete biological roles to collectively sustain immune suppression [117]. IL-6 activates STAT3 signalling in CD8^+^ T cells and worsens T-cell exhaustion status. Co-stimulation with IL-10 and TGF-β hampers NK-cell cytotoxic capacity through STAT3 and Smad2/3 signal activation. CCL22 functions as a chemoattractant, drawing CCR4-positive Treg populations into tumour sites. Meanwhile, IL-10 together with TGF-β boost Treg suppressive capacity via STAT5- and Smad3-dependent transcriptional programmes [118]. IL-6 and GM-CSF further drive STAT3-dependent MDSC expansion [119]. Expanded MDSC populations establish metabolic barriers by depleting tissue arginine and tryptophan. They additionally secrete ROS and NO to generate oxidative stress, which damages anti-tumour effector lymphocytes. Collectively, these signalling events establish self-amplifying feedback circuits that stabilise the immunosuppressive niche, facilitating tumour immune escape and blunting therapeutic responses to ICI-based treatments. It should also be noted that Wnt/β-catenin signalling, as depicted in Figure 3, represents another potent modulator of tumour-stroma-immune crosstalk reported across multiple malignancies. Nevertheless, concrete experimental evidence defining its immunomodulatory roles specifically within the NSCLC immune microenvironment remains limited, and further dedicated investigations are required to dissect its functional impact. Figure 3 illustrates the overall layout of this inter-cell communication network.

Single-cell and spatial technologies have begun to map how these populations organise inside human NSCLC lesions. Single-cell RNA sequencing of lung tumours has defined multiple myeloid and T-cell states and shown that their relative abundance varies widely between patients [19,49]. Spatial transcriptomic and imaging studies of lung adenocarcinoma have further shown that suppressive macrophages and dysfunctional T cells tend to occupy distinct niches within the tumour, and that the spatial organisation of these cells is linked to prognosis and to the response to immunotherapy [120]. Whether the proposed CEMIP-dependent interactions between tumour cells, macrophages and lymphocytes coexist in these niches has not yet been tested. No spatial or single-cell study has examined CEMIP expression in relation to immune cell states in NSCLC. This remains a gap that future studies could address directly.

Within this intercellular signalling network, CEMIP stands out as an interesting molecular node. In colorectal cancer models, CEMIP-derived hyaluronan fragments have been suggested to promote M2-like macrophage polarisation [35]. In breast cancer, CEMIP expression correlates with M2 macrophage infiltration [121]. Whether a similar STAT3-dependent pathway operates in NSCLC remains to be tested, since no direct evidence currently links CEMIP to monocyte-to-M2-TAM differentiation in this tumour type. If CEMIP-educated M2-like TAMs do exist, the soluble mediators they release could in principle impair CD8^+^ T-cell and NK-cell functions, but this step is speculative at present. Nevertheless, given its correlations with an immunosuppressive microenvironment, CEMIP remains a conceptually attractive target for interrupting suppressive crosstalk in NSCLC lesions (Figure 2). It should be noted that most parts of this mechanistic model are derived from pre-clinical laboratory data, whereas robust validation in human patient cohorts remains scarce. With such limitations taken into consideration, CEMIP still represents a worthwhile target to interfere with the mutually reinforcing suppressive cell-to-cell crosstalk present in NSCLC tumour tissue.

### 2.5. Summary Table of Key Immune Cell Subsets in the NSCLC TME

Table 1 summarizes the core phenotypic markers, major molecular regulators, key signalling pathways, and clinical significance of the main immune cell subsets in the NSCLC TME, providing a concise and comprehensive molecular perspective for understanding the immune cell landscape of the NSCLC TME.

### 2.6. Immunotherapy in NSCLC: Current Status, Resistance Mechanisms and Biomarkers

PD-(L)1 inhibitors are now standard treatment for advanced NSCLC, given as monotherapy or with platinum-based chemotherapy, and chemoimmunotherapy has moved into the adjuvant and peri-operative settings. A minority of patients achieves durable responses; most do not respond or eventually progress [6,54]. Resistance is multifactorial. Tumour-intrinsic features include HLA class I loss, IFN-γ pathway defects and Serine-threonine kinase 11 (STK11) or Kelch-like ECH-associated protein 1 (KEAP1) mutations. The TME matters equally: dense infiltration by M2-like TAMs, Tregs and MDSCs, together with exhausted CD8^+^ T cells, limits the efficacy of checkpoint inhibitors. These populations are discussed in Section 2.

Biomarker research has centred on PD-L1 expression, tumour mutational burden and immune-cell infiltration. PD-L1 tumour proportion score is the main clinically validated biomarker. Prognostic and predictive value must be separated: an association between immune-cell abundance and survival does not mean the marker predicts benefit from checkpoint inhibition [122]. The same distinction applies to the immune-cell markers in this review.

The immune-cell landscape in Section 2 thus provides the mechanistic background for the successes and failures of NSCLC immunotherapy. Directly targeting immune-cell populations remains experimental: CSF-1R inhibitors and similar agents are in clinical evaluation, whereas CEMIP-directed interventions remain at a preclinical, hypothesis-generating stage (Section 4.3).

## 3. Existing Research Deficiencies, Controversies and Limitations

Despite the substantial progress in elucidating the molecular mechanisms of immune cell subset regulation in the NSCLC TME in recent years, there are still many unresolved research deficiencies, controversies, and technical limitations in this field, which restrict the translation of basic research findings into clinical practice and the development of novel precise immunotherapeutic strategies.

### 3.1. Existing Research Deficiencies

(1) Insufficient research on immune cell subset heterogeneity at the single-cell level: Most current studies focus on the overall level of immune cell subsets in the NSCLC TME, and there is a lack of in-depth research on the heterogeneity of different subpopulations within the same immune cell subset at the single-cell transcriptome and proteome levels. For example, TAMs, CD8^+^ T cells, and Tregs all consist of multiple functionally distinct subpopulations [123], and their regulatory mechanisms and roles in the TME may be completely different. However, current studies have not clearly clarified the specific molecular characteristics and functional roles of each subpopulation in the NSCLC TME, which limits the development of precise targeted therapy strategies for specific immune cell subpopulations.

(2) Lack of research on the crosstalk between immune cells and other TME components: The NSCLC tumour microenvironment represents a complicated system containing immune cells [124], stromal cells, extracellular matrix, and metabolic components. A large body of current research concentrates on communication between different immune cell subsets. Yet many studies overlook other important regulators. These include extracellular matrix components such as hyaluronic acid and collagen [125], cancer-associated fibroblasts (CAFs), and metabolic factors including lactate and ketone bodies. Collectively, these factors can strongly influence immune-cell polarisation and functional performance, which is frequently under-appreciated in existing work. The lack of systematic research on the multi-component crosstalk of the TME leads to an incomplete understanding of the molecular regulatory network of the immunosuppressive TME.

(3) Insufficient clinical translation of basic research findings: Most research on the molecular mechanisms of immune cell regulation in the NSCLC TME is based on in vitro cell experiments and animal models, and there are few large-sample, multi-centre clinical studies to verify these findings. The results of in vitro and animal experiments cannot be fully translated into clinical practice due to the differences between the animal TME and the human TME, and the heterogeneity of human tumours, which affects the clinical application value of related research. In addition, there is a lack of research on the correlation between molecular regulatory factors (e.g., CEMIP) and clinical indicators (e.g., tumour stage, ICI efficacy), which restricts the development of novel molecular biomarkers and therapeutic targets.

### 3.2. Controversies in the Field

(1) Controversies in the role of B cells and TLS in the NSCLC TME: B cells and tumour-associated TLS represent one of the most debated topics within NSCLC tumour microenvironment research. Several reports regard TLS as a positive prognostic biomarker capable of boosting antitumour immune responses [126]. Nevertheless, other work fails to identify any prognostic benefit linked to TLS; in certain NSCLC subgroups, TLS even correlates with worse patient outcomes. These inconsistent findings can be partly explained by multiple sources of heterogeneity. Differences among B-cell subsets (effector B cells versus Bregs), TLS maturation status (immature versus mature), TLS location (intratumoural versus peritumoural), and NSCLC histological subtypes (adenocarcinoma and squamous cell carcinoma) may all account for such discrepancies. Larger-scale, multicentre clinical datasets combined with single-cell sequencing approaches will be required to resolve these open questions.

(2) Controversies in the prognostic and predictive value of immune cell subsets: Current research holds inconsistent views regarding whether immune cell subsets can serve as prognostic biomarkers or predictive indicators for ICI treatment in NSCLC. Many studies have reported that higher CD8^+^ T cell infiltration predicts better clinical outcomes in NSCLC patients [127]. However, relying solely on infiltration density is insufficient for precise prognosis and ICI response prediction. The actual predictive ability of CD8^+^ T cells is largely determined by their functional status, including exhaustion degree and inhibitory receptor expression. Moreover, there is still no unified and optimal strategy to combine various immune cell subsets with molecular markers such as tumour mutational burden (TMB), Microsatellite instability (MSI) and PD-L1 expression to improve predictive accuracy. Notably, experimental conclusions vary greatly among different research teams, leading to inconsistent research findings in this field.

(3) Controversies in the optimal target and scheme of immune cell-targeted therapy: Numerous immune-cell-directed treatment approaches have been assessed in preclinical work and clinical trials, including CSF-1R inhibitors for TAM modulation and CCR4-based agents aimed at Treg suppression. Even so, considerable uncertainty remains about which targets and combination regimens will work best for clinical application. As one example, CSF-1R inhibitors have demonstrated some clinical benefit within early-phase trials, yet these treatments come with notable limitations, including limited response rates and toxic effects such as hepatotoxicity and musculoskeletal injury. Pairing CSF-1R blockade with ICIs can enhance treatment outcomes [128], though key practical questions are still unresolved. It remains unclear what would be the ideal drug dosage, treatment sequencing, and suitable patient subgroups for such combinations. When it comes to Treg-depleting therapies targeting CD25, CTLA-4 or CCR4, target selection also stays debatable. Concerns mainly stem from the risk of broad-ranging systemic immune suppression triggered by these interventions.

### 3.3. Research Limitations

(1) Technical limitations of research models: Standard in vitro cell culture systems cannot fully replicate the complicated structural features, intercellular interactions, and dynamic metabolic alterations observed in the real NSCLC tumour microenvironment. As a result, data obtained from in vitro experiments often fail to reflect authentic in vivo tumour conditions. Common animal models, such as nude xenograft mice and genetically engineered mouse models (GEMMs), are widely used to simulate the NSCLC TME in preclinical research [129]. Even so, key differences in immune composition and microbial flora exist between murine and human tumour microenvironments. Such interspecies differences greatly hinder the clinical transformation of existing research findings. At present, the insufficient availability of high-fidelity humanized mouse models and tumour organoid systems remains a major technical bottleneck, restricting accurate simulation of the native human NSCLC TME.

(2) Lack of standardised detection methods for immune cell subsets: At present, the detection methods of immune cell subsets in NSCLC tissues include flow cytometry, immunohistochemistry (IHC), immunofluorescence, and single-cell sequencing, but there is no unified national or international detection standard. The differences in marker selection (e.g., different M2 TAM markers), sample processing methods (e.g., fresh tissue vs. paraffin-embedded tissue), and result judgment standards (e.g., positive cell ratio vs. staining intensity) between different laboratories lead to the difficulty in horizontal comparison of research results, which affects the systematic summary and meta-analysis of research data.

(3) Critical Evaluation of the Proposed CEMIP–STAT3 Axis: Research on CEMIP in the NSCLC immune microenvironment remains at a hypothesis-generating stage. We have therefore labelled each claim with one of four evidence levels (Table 2): human NSCLC data, NSCLC animal models, NSCLC in vitro experiments, and cross-tumour or indirect evidence. The NSCLC-specific evidence covers tumour-cell-intrinsic functions (expression, prognosis, proliferation, migration/EMT and chemoresistance) [36,38,39,40] and a correlational link to immunosuppressive infiltration [12]. Every step of the proposed CEMIP–STAT3–TAM–effector cascade, by contrast, rests on cross-tumour evidence or inference. CEMIP–STAT3 coupling has been shown in breast cancer but not in the lung [14]; CEMIP effects on macrophages come from glioblastoma and brain-metastasis models [60,61]; and STAT3-driven M2 polarisation is well established in macrophages. The monocyte/macrophage receptor for secreted CEMIP has not been identified, and CEMIP-dependent CD8^+^ T or NK dysfunction has not been demonstrated. These gaps limit how far the conclusions of this section can be pushed.

Alternative mechanisms deserve consideration before a direct CEMIP to receptor to STAT3 cascade is assumed. CEMIP may act through its enzymatic product: hyaluronan fragments signal via TLR2/TLR4 and CD44 and can re-educate macrophages without a CEMIP-specific receptor [13]. CEMIP-driven ECM remodelling may change immune cell trafficking within the tumour [12,35]. Its immune effects may also be a by-product of tumour-cell-intrinsic oncogenic signalling, since CEMIP stabilises EGFR [40]. TAM-expressed CEMIP may matter independently of tumour-cell secretion, which has been reported in glioblastoma [61]. These mechanisms are not mutually exclusive and would not all point to the same therapeutic strategy.

A related question is whether CEMIP adds non-redundantly to STAT3 activation. STAT3 is constitutively active in a large fraction of human NSCLC and is maintained by IL-6/JAK1 [130], JAK2/gp130 [131] and EGFR signalling [132]; in STK11-mutant NSCLC, myeloid STAT3 has been linked to resistance to PD-(L)1 blockade [133]. In KRAS-mutant lung adenocarcinoma, STAT3 can even act as a tumour suppressor by retaining NF-κB in the cytoplasm and limiting IL-8-driven myeloid infiltration [134]. Proving non-redundancy therefore requires loss-of-function data: CEMIP deletion or blockade should measurably change STAT3 phosphorylation, TAM polarisation and effector T/NK function beyond what IL-6/JAK and EGFR signalling can explain.

The experiments needed are clear in outline: co-culture or supernatant-transfer experiments with primary human monocytes/macrophages and CEMIP-knockout or CEMIP-overexpressing NSCLC cells (phospho-STAT3 and M2 markers as readouts); receptor identification by unbiased interaction screens, or evidence that hyaluronan-fragment signalling fully accounts for the effects; syngeneic NSCLC models with CEMIP deleted in tumour cells or myeloid cells; and NSCLC patient cohorts with matched immune phenotyping. Until such data exist, the CEMIP–STAT3–TAM axis should be regarded as a candidate mechanism rather than an established one.

## 4. Future Research Directions and Prospects

Advances across high-throughput sequencing, single-cell analytical platforms and gene-editing tools have reshaped how researchers characterise immune-cell landscapes within the NSCLC tumour microenvironment. Nevertheless, multiple knowledge gaps, conflicting experimental observations and technical bottlenecks still restrict the transfer of laboratory-derived results into clinical practice. Against this background, several priority research avenues are discussed below, covering mechanistic exploration, clinical-translation efforts and the exploitation of innovative therapeutic targets.

### 4.1. In-Depth Research on the Heterogeneity of Immune Cell Subsets at the Single-Cell Level

Immune-cell heterogeneity remains a major stumbling-block when trying to unpack NSCLC tumour-microenvironment biology. Even though we now have access to powerful experimental tools such as single-cell RNA sequencing (scRNA-seq), single-cell ATAC sequencing (scATAC-seq) and single-cell proteomics, many published analyses still stop at broad immune-cell categories, without digging deep into discrete sub-populations. In practice, single-cell multi-omics workflows give us an opportunity to tease apart different subgroups belonging to TAMs, CD8^+^ T cells, Tregs and MDSCs from patient tumour samples. Rather than simply cataloguing molecular differences, we need to map out their transcriptional wiring and actual functional behaviours, so we can tell which subsets are truly driving local immune suppression and poor responses to ICIs.

If we look at tumour-associated macrophages as a concrete example, conventional bulk sequencing data has major limitations here. It is difficult to separate pro-tumour M2-biased macrophages from antitumour M1-type populations using bulk-level analysis alone [135]. Single-cell sequencing techniques offer an opportunity to uncover distinct surface markers and downstream signalling features that define these tumour-promoting macrophage subgroups.

Most existing single-cell datasets for NSCLC are built from static tumour biopsies taken at one single time-point. This leaves us missing critical information about how immune compartments shift over the course of immunotherapy. Collecting serial patient samples, before ICI initiation and throughout treatment, would fill this gap. Following dynamic immune-cell remodelling across treatment timepoints could shed light on the real-world origins of acquired ICI resistance, though patient recruitment and repeated tissue sampling bring clear practical clinical hurdles.

### 4.2. Elucidate the Multi-Component Crosstalk Molecular Network of the NSCLC TME

The NSCLC TME is a complex ecosystem composed of multiple components, and the functional regulation of immune cells is the result of the joint action of immune cells, stromal cells, extracellular matrix, and metabolic factors [136]. Future research should focus on the crosstalk between immune cell subsets and other TME components, and construct a comprehensive multi-component crosstalk molecular network of the NSCLC TME. For example, explore the regulatory role of cancer-associated fibroblasts (CAFs) in TAM polarisation and CD8^+^ T cell exhaustion via the secretion of cytokines (e.g., IL-6, TGF-β) and extracellular matrix components (e.g., periostin); clarify the effect of metabolic factors in the TME (e.g., lactic acid, ketone bodies, adenosine) on the phenotypic transformation and functional regulation of immune cells via metabolic signalling pathways (e.g., GPR81, mTOR); and study the interaction between the microbial flora and immune cell subsets in the NSCLC TME, and its regulatory role in the anti-tumour immune response.

### 4.3. Targeting CEMIP: An Emerging Research Direction to Re-Educate the NSCLC TME

CEMIP has been proposed as an emerging tumour-derived regulator of the immunosuppressive NSCLC TME, although direct evidence linking it to STAT3 signalling in lung cancer is still lacking. Future research should focus on the in-depth exploration of the molecular regulatory mechanism of CEMIP and its translational potential as a novel therapeutic target. The mechanistic framework underlying these opportunities is summarised in Figure 2. Research directions mainly include the following:

Clarify the specific molecular mechanism of CEMIP regulating TAM polarisation: Identify the specific surface receptor on monocytes/macrophages bound by CEMIP using co-immunoprecipitation, surface plasmon resonance (SPR), and other technologies; elucidate the downstream transcriptional regulatory network of the CEMIP/STAT3 signalling pathway using chromatin immunoprecipitation sequencing (ChIP-seq) and RNA sequencing (RNA-seq) technologies, and identify the key target genes regulating M2 TAM polarisation.

Explore the direct regulatory effect of CEMIP on other immune cell subsets: Verify whether CEMIP can directly bind to the surface of CD8^+^ T cells, NK cells, and Tregs and regulate their function via specific signalling pathways using in vitro cell co-culture and in vivo animal experiments; clarify the comprehensive regulatory role of CEMIP in the immune cell crosstalk network of the NSCLC TME.

Develop CEMIP-targeted reagents in an exploratory, preclinical context: no CEMIP-specific small molecule inhibitors, neutralizing antibodies or siRNA-based agents have been developed to date. Such reagents would first need to be generated on the basis of the CEMIP structure and tested for their ability to reverse the immunosuppressive TME and enhance anti-tumour immunity in mouse models of NSCLC. Only after these preclinical steps have been completed could combinations of CEMIP inhibition with ICIs, chemotherapy or radiotherapy be explored, and any clinical application remains purely hypothetical at this stage.

Verify the clinical significance of CEMIP: We can measure CEMIP expression across large NSCLC tissue cohorts using IHC and quantitative reverse-transcription PCR (qRT-PCR). It would then be worthwhile to analyse how CEMIP levels correlate with patients’ clinicopathological features, clinical outcomes and responses to immune checkpoint therapy. Findings from such studies may help to explore whether CEMIP could potentially serve as a candidate biomarker for prognosis and ICI-response prediction in NSCLC, given that supporting clinical evidence remains limited at present.

Several pre-clinical tumour models have explored drug approaches acting on upstream components within the CEMIP-STAT3-centred immunosuppressive network. Beyond small-molecule STAT3-targeting agents, antibody-based therapeutics interfering with upstream STAT3-activating cytokines are also under early-phase clinical evaluation. For instance, anti-LIF monoclonal antibody MSC-1 (AZD0171) has completed a phase-I trial for advanced solid malignancies; LIF-LIFR signalling represents one upstream axis capable of triggering intracellular STAT3 activation in myeloid cells, though its immunomodulatory effects remain to be fully characterised in NSCLC settings [119]. Repurposed CSF-1R inhibitors are capable of restraining monocyte-to-M2-macrophage reprogramming [43]. Theyare now being assessed together with immune checkpoint blockade in early-phase lung-cancer clinical trials, though they do not directly interfere with CEMIP-related signalling. To date, no specific CEMIP-targeted small-molecule inhibitors or antibody-based therapeutics are available for clinical application in NSCLC. Further pre-clinical optimisation is required to improve target specificity, delivery and tolerability before translating these promising molecular targets into novel anticancer agents for clinical practice.

### 4.4. Establish Standardised Detection Methods and Develop Multi-Biomarker Prediction Models

Insufficient standardisation for immune-cell-subset measurement constitutes one practical barrier limiting clinical adoption of immune-related biomarkers. Consensus-driven global testing specifications are therefore required for analysing immune populations within NSCLC specimens. Such standards need to cover marker selection protocols, unified sample-handling workflows and objective result-evaluation rules, so that experimental outputs generated from separate laboratories become more comparable and reproducible. Once such standard operating procedures become available, researchers may integrate immune-cell read-outs such as M2-TAM abundance and CD8^+^ T-cell exhaustion status alongside molecular markers including TMB, MSI, PD-L1 and CEMIP expression [137]. Machine-learning and AI-driven algorithms can then support building multi-marker predictive panels. These integrated models aim to improve the accuracy of prognosis and ICI-response prediction. Besides tissue-based detection, liquid-biopsy approaches targeting circulating immune entities such as circulating tumour-associated macrophages (CTAMs) and exhausted circulating CD8^+^ T cells deserve further development. These peripheral-blood-oriented assays support non-invasive, real-time tracking of TME immune status and carry considerable clinical utility.

### 4.5. Promote the Clinical Transformation of Immune Cell-Targeted Combined Immunotherapy

Immune cell-targeted therapy combined with ICIs is the main development direction of NSCLC immunotherapy in the future. To promote the clinical transformation of these therapeutic strategies, future research should focus on the following aspects:

Carry out large-sample, multi-centre phase II/III clinical trials: On the basis of promising preclinical and early-phase clinical trial results, carry out large-sample, multi-centre, randomized, controlled phase II/III clinical trials for immune cell-targeted therapeutic strategies (e.g., CSF-1R inhibitors, CCR4 inhibitors) combined with ICIs, to verify their efficacy and safety in the treatment of advanced NSCLC.

Explore the optimal therapeutic scheme and applicable population: Clarify the optimal drug dose, administration sequence, and treatment course of combined therapy through clinical trials; identify the specific population suitable for combined therapy using the multi-biomarker prediction model, to avoid ineffective treatment and reduce adverse reactions.

Develop novel immune cell-targeted drugs with high efficiency and low toxicity: Improve the targeting and efficacy of immune cell-targeted drugs using antibody-drug conjugates (ADCs), bispecific antibodies, and nanoparticle drug delivery systems; reduce the systemic adverse reactions of drugs by targeting the tumour microenvironment (e.g., hypoxia-responsive, pH-responsive drug delivery systems).

Combine immune cell-targeted therapy with other therapeutic modalities: Future work ought to examine how immune-cell-directed therapies perform when paired with other established NSCLC treatment modalities, such as chemotherapy, radiotherapy, anti-angiogenic drugs, and molecular-targeted agents represented by EGFR-TKIs and ALK inhibitors. It is still not fully understood how these different treatment approaches produce synergistic effects. Besides, there is no consensus regarding the most suitable matching patterns and administration protocols for combined use. Additional research is therefore required to dissect potential synergistic mechanisms and work out reasonable combination strategies for NSCLC.

## 5. Conclusions

Globally, NSCLC accounts for higher mortality burden compared with many other cancer forms. The tumour-suppressive microenvironment is central to tumour immune escape and the emergence of ICI resistance, which may present as primary refractoriness or develop after an initial therapeutic response. NSCLC lesions contain a diverse mix of immune cells. Key populations include TAMs, CD8^+^ cytotoxic T cells, Tregs, NK cells and MDSCs. These cells rarely act alone. They interact through cytokines, chemokines and metabolic mediators. Together they form an interconnected immunosuppressive network. This network shapes tumour progression. It also determines the response to therapy [3].

Functional defects across these immune compartments, most notably M2-skewed TAM polarisation, CD8^+^ T-cell exhaustion, impaired NK-cell activity and excessive Treg/MDSC accumulation, are major forces that establish the NSCLC immunosuppressive TME. Crosstalk among these functionally altered immune populations works cooperatively to dampen anti-tumour immunity, facilitate tumour-cell proliferation, local invasion and distant metastasis, and consequently bring about unfavourable patient prognosis and ICI treatment failure [122]. CEMIP abundantly produced in NSCLC tissues, acts as a valuable molecular bridge within this immune-cell-communication network [37]. Inhibitory-receptor expression rises along with this dysfunction. This positions the CEMIP-STAT3 axis as one candidate upstream modulator of immune-checkpoint-associated immune evasion in NSCLC. By triggering STAT3-driven M2-TAM polarisation, CEMIP indirectly alters CD8^+^ T-cell and NK-cell function through immunosuppressive products released by reprogrammed TAMs. This property renders CEMIP a promising molecular candidate for interventions aiming to reverse NSCLC-associated immunosuppression. Nevertheless, it is critical to interpret such pre-clinical findings with caution. Current understanding of CEMIP-mediated immune regulation in NSCLC is built largely on cell and animal experimental data. Robust evidence from large-sample human NSCLC cohorts is still missing, and CEMIP has not been validated for clinical biomarker or therapeutic usage in patients.

This review focuses on progress made in the last three to five years, and also draws on older studies where they provide the mechanistic foundation that current concepts build on. It summarises the molecular regulation of major immune subsets in the NSCLC TME. We integrate current understanding of intercellular signalling that sustains this suppressive network, and illustrate how immune-cell dysfunction bears clinical meaning for prognosis as well as ICI treatment outcomes. We further outline outstanding knowledge gaps (for instance incomplete appreciation of immune-cell heterogeneity, limited data on multi-component TME crosstalk) and, ongoing field-wide debates (including dual biological roles of B cells and TLS, prognostic performance of immune-cell markers), together with practical technical constraints such as imperfect pre-clinical models and non-uniform detection workflows. Based on these points, we put forward corresponding avenues for follow-up investigation.

Overall, interfering with immune-cell-specific signalling axes and breaking suppressive inter-cellular crosstalk within the TME, when combined with ICIs and other anti-tumour modalities, represents a meaningful therapeutic direction for advanced NSCLC. Substantial obstacles remain when translating pre-clinical discoveries into real-world clinical practice. Even so, joint efforts from both basic-science investigators and clinical oncologists are expected to drive further progress in the study of NSCLC TME immune landscapes and deliver improved treatment options for affected patients.

## Figures and Tables

**Table 1:** Key immune cell subsets in the NSCLC tumour microenvironment: phenotypic markers, molecular regulators, signalling pathways and clinical significance.

Immune Cell Subset	Key Phenotypic Markers	Major Molecular Regulators	Key Signalling Pathways	Clinical Significance	Evidence Source/Strength
M2 TAMs	CD206, Arg-1, IL-10, CD163	CEMIP, CSF-1, CCL2, IL-4/13, HIF-1α	STAT3, PI3K/Akt, HIF-1α, JAK/STAT6	Poor prognosis, ICI resistance, independent prognostic marker	Human NSCLC cohorts + clinical association
Exhausted CD8^+^ T cells	PD-1, TIM3, LAG3, TIGIT	Chronic antigen, IL-6, IL-10, TGF-β, TOX	STAT3, NFAT, TOX/NR4A1, Smad3	Impaired anti-tumour immunity, ICI resistance	Human NSCLC (single-cell) + in vitro
Tregs	Foxp3, CD25, CTLA-4, GITR	CCL22, TGF-β, IL-2, lactic acid	STAT5, Smad3, PI3K/Akt, GPR81/ERK	Poor prognosis, ICI resistance, tumour stage correlation	Human NSCLC + clinical association
Dysfunctional NK cells	NKG2D ↓, KIR ↑, TIGIT ↑, PD-1 ↑	IL-10, TGF-β, ROS, NO, MICA/B↓	STAT3, Smad2/3, MAPK/ERK, NKG2D/DAP10	Poor prognosis, tumour metastasis	In vitro + animal models + clinical association
MDSCs (G-MDSCs/M-MDSCs)	CD11b+CD33+HLA-DRlow, CD15+/CD14+	GM-CSF, G-CSF, IL-6, PGE2, CCL2	STAT3, NF-κB, NADPH oxidase, iNOS	Poor prognosis, ICI resistance, potential prognostic marker	Human NSCLC + in vitro + animal models
γδ T cells	TCRγδ, NKG2D, CD3	Phosphoantigens, MICA/B, IL-15	NKG2D/DAP10, TCR/ERK, JAK/STAT5	Favorable prognosis, anti-tumour immunity	Human NSCLC (correlational) + clinical studies
B cells/TLS	CD19, CD20, CXCR5, IgD	CXCL13, LTα1β2, IL-4, IFN-γ	CXCR5/CXCL13, LTβR, JAK/STAT6	Favorable prognosis (TLS), immune suppression (Bregs)	Human NSCLC + clinical association

**Table 2:** Summary of the evidence supporting the proposed CEMIP–STAT3–TAM axis in NSCLC.

Evidence Level	Tumour Type	Experimental Model	CEMIP Manipulation	Affected Immune Population	Main Finding	Independent Validation
L1 (human NSCLC)	NSCLC	Human tumour specimens (IHC, *n* = 153)	None (expression/prognosis)	–	Strong CEMIP staining in 49.67% of tumours; association with poor differentiation, lymph-node metastasis, advanced stage and shorter OS; independent prognostic factor (*p* = 0.040)	Consistent across cohorts
L1 (human NSCLC)	NSCLC/LUAD	Human tissue + databases	None (expression)	–	mRNA and protein upregulation; independent hazard ratio 1.833	Independent models
L1 (human NSCLC, correlational)	LUAD	Human tissue + databases	None (expression correlation)	TAMs, M2-like TAMs, Tregs, exhausted CD8^+^ T cells	CEMIP correlates with immunosuppressive infiltration markers (CCL2, CD68, IL-10; CD163, VSIG4, MS4A4A; FOXP3, CCR8, STAT5B; CTLA-4, TIM-3)	Database cross-validation; association only
L1 (human NSCLC)	NSCLC	Human tumour specimens (*n* = 176)	None (pEGFR–pSTAT3 correlation)	–	pEGFR–pSTAT3 strongly correlated; pSTAT3 linked to reduced apoptosis	–
L1 (human NSCLC, biomarker)	Lung cancer	Patient plasma EVs	None (detection)	–	CEMIP detectable in EV fraction; higher in patients with brain metastases	Single-centre
L2 (animal)	NSCLC	Xenograft model	Knockdown	–	Reduced tumour growth; improved chemotherapy response	Two studies
L2 (animal, context)	LUAD (KRAS)	GEMM	STAT3 disruption (comparator)	Myeloid cells	STAT3 suppresses tumourigenesis: NF-κB retained in cytoplasm, reduced IL-8-driven myeloid infiltration	–
L3 (in vitro)	NSCLC	Cell lines	Knockdown/overexpression	–	EMT via PI3K-Akt; migration and invasion	Two independent studies
L3 (in vitro)	NSCLC	Paclitaxel-resistant cells	ALKBH5–CEMIP axis	–	EMT and chemoresistance	–
L3 (in vitro)	NSCLC	Osimertinib-resistant models	Transcriptional circuit (incl. CEMIP)	–	Non-canonical core regulatory circuit drives resistance	–
L3 (in vitro, context)	NSCLC	Cell lines	IL-6 neutralisation / JAK1 (comparator)	–	JAK1–STAT3 phosphorylation suppressed by IL-6 blockade	–
L4 (other tumour, indirect)	Breast cancer (brain metastasis)	In vivo models + cells	CEMIP^+^ exosomes	Microglia, brain endothelium	Pro-inflammatory programme (Ptgs2, Tnf, Ccl, Cxcl); metastatic niche formation	Independent models
L4 (other tumour, indirect)	Glioblastoma	Cell lines + HYBID-KO mice	HYBID knockdown/KO	Macrophages	Reduced growth; reduced macrophage infiltration and pro-tumour factor release	KO mice + in vitro
L4 (other tumour)	Non-lung systems	Cell systems	Overexpression/knockdown	–	NF-κB-induced CEMIP protects EGFR from degradation	–
L4 (other tumour)	Breast cancer	Cell lines	CEMIP manipulation	–	GRP78–STAT3 axis promotes proliferation and migration	–

## Data Availability

No new data were created or analyzed in this study. Data sharing is not applicable to this article.
